# Holistic application of the one health approach in the prevention and control of rabies: plausible steps towards achieving the 2030 vision in Africa

**DOI:** 10.1186/s42522-024-00108-6

**Published:** 2024-09-11

**Authors:** Olalekan Chris Akinsulie, Oluwawemimo Oluseun Adebowale, Ridwan Olamilekan Adesola, Olamilekan Gabriel Banwo, Ibrahim Idris, Seto Charles Ogunleye, Oluwole Fasakin, Adetolase Bakre, Ifeoluwa Peace Oladapo, Victor Ayodele Aliyu, Emily Onesai Waniwa, Oluwatobi Fasiku, Melina Joshi, Mercy Olorunshola

**Affiliations:** 1 Department of Veterinary Microbiology and Pathology, Washington State University, Pullman, WA USA; 2 College of Veterinary Medicine, Federal University of Agriculture Abeokuta, Abeokuta, Ogun State Nigeria; 3 Department of Veterinary Medicine, Faculty of Veterinary Medicine, University of Ibadan, Ibadan, Nigeria; 4 Department of Veterinary Medicine, Faculty of Veterinary Medicine, Usman Danfodiyo University, Sokoto, Nigeria; 5 Comparative Biomedical Sciences, Mississippi State University, Mississippi State, Starkville, MS 39760 USA; 6 Crintex Solution Limited, Port Harcourt, Nigeria; 7 Central Veterinary Laboratory, Division of Veterinary Technical Services, Ministry of Lands, Agriculture, Water and Rural Resettlement, Harare, Zimbabwe; 8 Center for Molecular Dynamics Nepal, Kathmandu, Nepal; 9 Department of Biological Sciences, State University of New York at Binghamton, Binghamton, NY USA

**Keywords:** Africa, One Health, Rabies, 2030 Eradication

## Abstract

Rabies remains a significant public health challenge in Africa, primarily burdening impoverished rural communities, with children and young adults being the most vulnerable. Achieving complete elimination in the continent by 2030 requires a coordinated effort hinged on the One Health concept, external support from international organizations like the World Health Organization (WHO) and the national governments of endemic countries. Here, we reviewed the various socio-economic and ecological factors influencing the spatial distribution and molecular epidemiology of the disease. To mitigate the transmission of rabies on a global scale, and specifically in Africa, we proposed a multi-pronged approach including enhanced access to healthcare resources, cultural sensitization and massive health promotion with efforts geared towards promoting responsible dog and pet ownership and population management, effective monitoring, and mitigation of environmental changes.

## Introduction

Rabies is a vaccine-preventable, neglected tropical disease (NTD) and the deadliest viral zoonosis known in history. It is highly fatal (100%) in humans and animals once symptoms appear. Over 99% of human rabies reported are dog – mediated [1, 2], though this disease has been reported in other domestic and wild animals such as bats [3, 4]. Wildlife transmission of rabies to humans are negligible. Rabies virus (RABV) in the saliva of infected dogs is transmitted to humans usually through bites, scratches or direct contact with mucosa with eyes, mouth or open wounds [5]. The disease can be efficiently prevented and controlled through improved political commitment, mass vaccination of dogs, enhanced accessibility of risk or vulnerable groups (especially rural communities and children) to Pre-exposure prophylaxis (PrE) and post-exposure prophylaxis (PEP), implementation and enforcement of responsible dog ownership, prevention of dog bites and stray dogs, and effective surveillance systems for early detection and response [6].

**Fig. 1:**
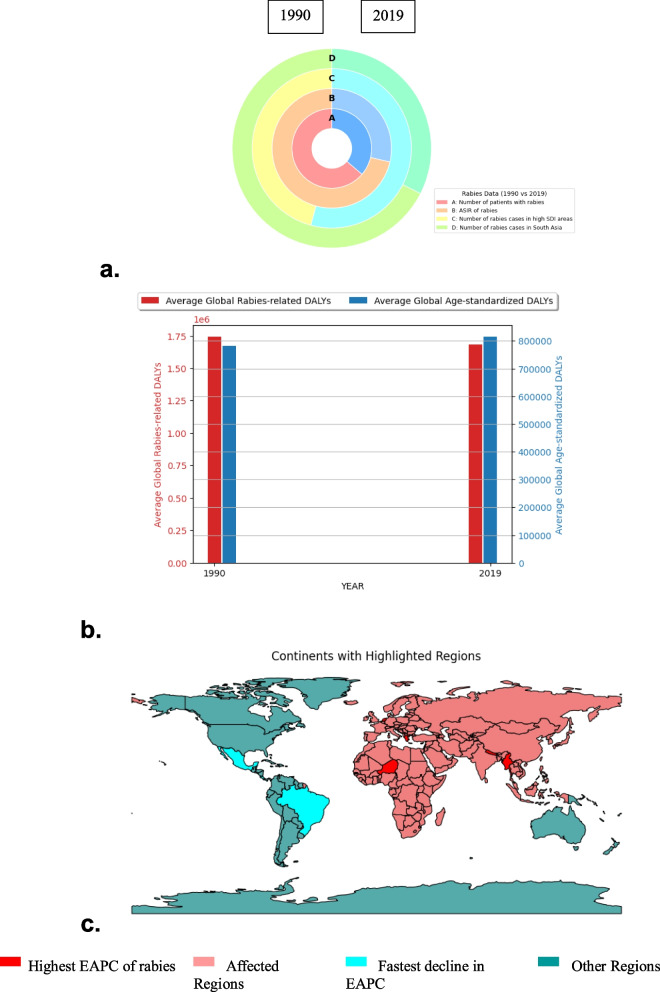
Schematic representation of the global distribution of Rabies between 1990 and 2019. Globally, over the last 3 decades, the incident cases of rabies have significantly reduced to half its estimate in 1990 (**a**). A progressive improvement and bolstering of the sociodemographic index (SDI) have positively impacted the incidence and mortality rates. Moreover, the disability-adjusted life years (DALYs) of rabies showed over 50% decline in estimation in 2019 compared to 1990 (**b**). The estimated annual percentage change (EAPC) of rabies cases has varying levels across continents (**c**). Adapted from Gan H et. al., 2023

Rabies is widely distributed globally except in Australia and Antarctica where no cases of dog mediated rabies have been reported as shown in Fig. 1 [5, 7, 8]. In addition, rabies mediated by dog bites has been eliminated from western Europe, Canada, the USA, Japan, and Latin American countries [9, 10]. Nevertheless, rabies is still a challenging health issue in many Asian and African countries with tens of thousands of human deaths occurring every year [5]. Rabies have devastating human, social and economic burden especially on the poor, vulnerable and marginalized populations. The disease has huge consequences on achieving the Sustainable Development Goals (SDGs) 1, 3, 10 and 17. It has been estimated that 50,000–60,000 human deaths occur every year globally, with 95% deaths in Asia and Africa, and 40% of children under 15 years of age affected [5].

Economically, rabies causes an estimated cost of US$8.6 billion per year globally, while expenditure for rabies control and prevention in Asia and Africa is estimated to exceed US$500 million, with most of these costs spent on provision of PEP [11]. A study conducted in Tanzania showed that an average patient in rural areas where people live on less than US$1 per day, would need to spend over US$100 to complete WHO recommended PEP schedules [11]. The associated high costs of disease prevention, lack of PEP, patronization of non-health facilities have related to high death rates in Africa. In recent times, the dynamics between the interaction of humans, animals, and their environment in has multiplied the risk of zoonoses emergence and re-emergence, and the spread at national, regional, or international levels. These global health challenges have prompted the need to invent a strategy that addresses disease risks wholistically via considering more broadly the complexity of human health and its linkages with surrounding environment and the health of animals. This review emphasizes a holistic strategy for effectively implementing integrated health systems to enhance rabies prevention in Africa. It identifies key areas of focus and proposes practical methods to achieve the WHO vision of eradicating RABV and preventing human deaths by dog-mediated rabies by 2023.

### Transmission

Various mammals, including dogs, foxes, wolves, jackals, raccoons, skunks, mongoose, and numerous species of bats, can serve as reservoir hosts of rabies virus that then transmit the virus to other mammals [12]. Rabies virus can infect most mammals, with a high degree of mortality. Although the disease occurs extensively in Africa, rabies is estimated to cause approximately 60,000 human deaths worldwide annually, 40% of whom are children living in Africa and Asia. Regardless of the vaccination efforts, most human rabies cases still result from bites of rabid dogs in Africa and Asia. In North America, bats have been the source of most human rabies cases [13]. There are 3 major modes of RABV spread to humans including bites, mucous membrane exposure, and, less commonly, aerosol inhalation. Rabies virus-laden saliva is inoculated through the skin into the muscle and subcutaneous tissues of the victim following the bite of a rabid animal, and this is the most common mode of transmission and infection. Most infections are transmitted by dog bites, owing to their close association with humans. Usually, an intact skin is impervious to the virus. Scratches are required, even though scratches laden with infectious saliva are a less common transmission source, as the infection risk is several folds less than actual bites. Subcutaneous infection may occur during skin exposure following a small bite that escapes attention. However, there is still a dearth of clear information about the mode of viral entry from dermal nerves into the CNS by the bat transmitted RABV [14]. Aerosolized RABV transmitted via inhalation had been linked to an accidental exposure of a laboratory worker to RABV while homogenizing goat brains for rabies vaccine production and by people near caves where numerous infected bats live [15]. Human-to-human transmission is uncommon, and every known case is iatrogenic following tissue transplantation of organs harvested from undiagnosed, rabies-infected donors. Following inoculation of RABV into muscle and subcutaneous tissues, the virus binds to nicotinic acetylcholine receptors (nAChR) at the neuromuscular junction and progresses toward the spinal cord within axons of peripheral nerves by retrograde fast axonal transport [14]. The dissemination of RABV occurs within the CNS axons, using multiple routes to reach the motor or sensory neurons of the CNS, largely determined by the site of inoculation and post-infection time [16]. Consequently, there is centrifugal spread from the CNS through the nerves to multiple organs, including the salivary glands of the transmitting animals beginning the cycle all over [17]).

### Molecular epidemiology and phylogeny of human and animal rabies in Africa

**Fig. 2:**
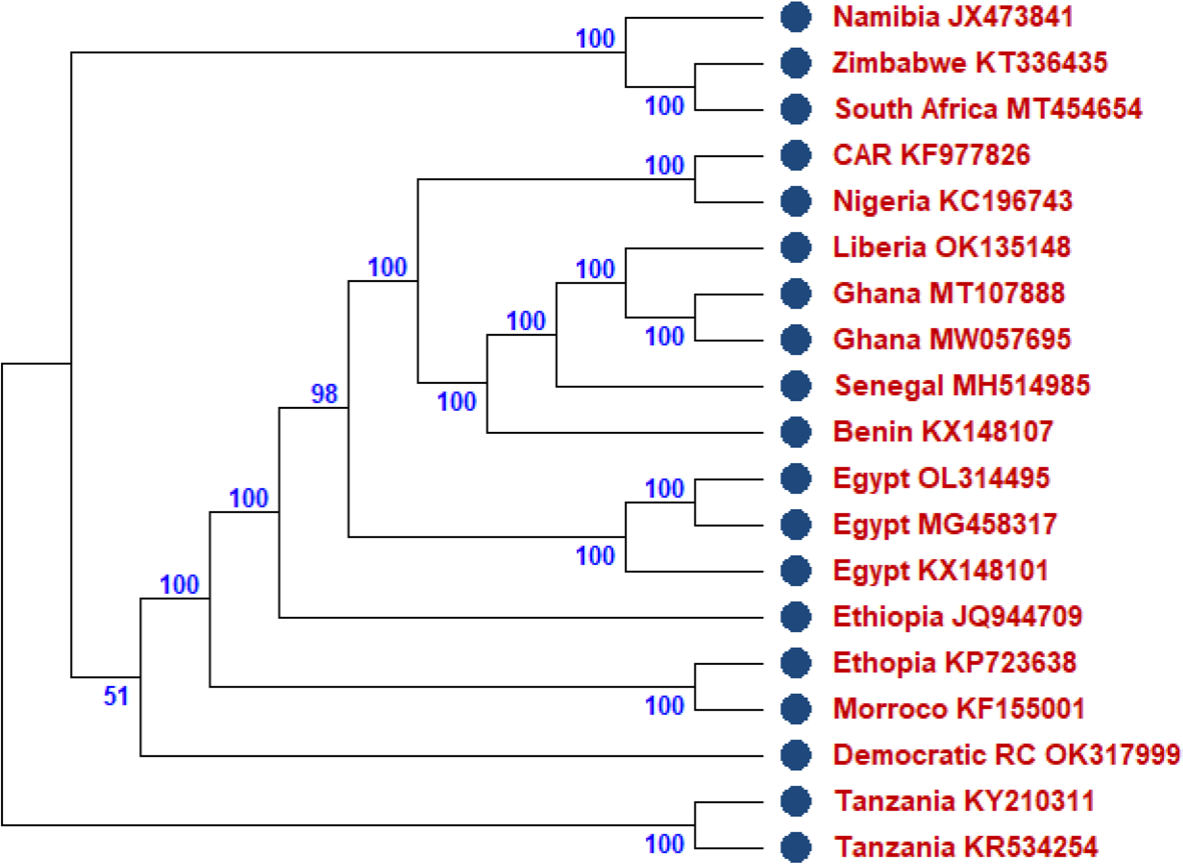
Phylogenetic tree showing rabies virus genotypes from Africa. The tree was generated using maximum likelihood reconstruction by the general time reversible model with a gamma distribution and proportion of invariable sites (GTR+I+G). The analyses were based on whole genome sequences with 11923 nucleotides long. The sequences are labeled with the country names and accession numbers

Six large, well-characterized clades of Rabies virus have been identified within the divergent dog-specific cluster [18]. These clades are referred to as the African-2, African-3, Artic-related, Asian, Cosmopolitan, and Indian clades. According to studies, bat-specific RABV is primarily found among bats originating in the New World [19]. Molecular epidemiology studies of RABV in Africa revealed the presence of these six clades [20]. These clades all contain the classical RABV species, which can be further divided into several subclades and lineages according to geographic location, virus variability, and reservoir hosts [21, 22]. Field RABV isolates of the Cosmopolitan clade can be divided into two main subclades, Africa-1 and Africa-4 [23]. Africa-1 (Africa-1a and 1b) subclade has been demonstrated to circulate in the northern, eastern, and southern sections of Africa [24], whereas Africa-4 has recently been discovered in northern Africa [25]. It has been demonstrated that the Africa-1 and Africa-2 lineages co-circulate in Nigeria and the Central African Republic [23, 26]. The Africa-2 lineages are continuously found throughout West and Central Africa. Table 1 shows the various African countries where the RABV whole genome sequences have been isolated and corresponding animals. Domestic dogs are essentially the only population necessary for preserving canid variations in various parts of Africa, even though Africa-1 and Africa-2 lineages have been recorded in several domestic and wild carnivore species [27]. On the other hand, it has been hypothesized that wild canids help maintain canine rabies cycles in particular regions of South Africa, Namibia, and Zimbabwe [28]. Within viverrid animals in southern Africa, the third Africa-3 clade—which is well suited to mongooses—is maintained through a separate epidemiological cycle from the canine RABV [27]. Comprehensive molecular epidemiology of RABV among human and animal populations has not yet been established, despite the fact that Rabies cases have been continually reported throughout Africa [29]. As shown in Fig. 2, the phylogenetic analysis of nucleotide sequences from African countries that the RABV viruses originating from Zimbabwe, South Africa, Namibia, Tanzania, Democratic Republic of Congo, Ethiopia, Morocco, and Egypt, (Clade 1) were genetically distinguishable from those obtained from Central African Republic, Nigeria, Benin, Senegal, Liberia, Ghana (Clade 2). Notably, these clusters represent a new phylogenetic group among others. The sequences were isolated from a wide range of hosts. No report about whole genome sequences isolated from bats in Africa was found. Apart from the in-country similar sequences, the Zimbabwe sequence showed similarity with sequences from South Africa and Namibia, as well as a sequence from the Democratic Republic of Congo and Tanzania. Similar pattern was seen in sequences from Morocco and Ethiopia. This might be a result of these countries sharing borders with one another, causing cross-border transmission of RABV in the same clade (Clade 1) [30]. In Clade 2 of the phylogenetic tree, the sequence from Central African Republic is closely related to the sequence from Nigeria. Although sequences from Benin, Senegal, Liberia, Nigeria, and Central African Republic are slightly different from Ghana sequences, it appears that distinct strains of RABV are ravaging these countries.

**Table 1:** African countries where whole genome sequences of Rabies virus have been 166 isolated and their corresponding hosts

**Countries of Isolation**	**Hosts**
Zimbabwe	Dogs and Jackals
South Africa	Jackal
Namibia	Kudu
Tanzania	Dogs and Cat
Democratic Republic of Congo	Dog
Ethiopia	Jackal
Morocco	Cow
Egypt	Human, and Dog
Central African Republic	Human
Nigeria	Dog
Benin	Cat
Senegal	Wolf
Liberia	Dog
Ghana	Humans, and Dog

### Methodology for the phylogenetic analysis

A total of 19 whole genome sequences with 11,923 nucleotides long were downloaded from GenBank. These sequences were isolated from 14 African countries. Countries with partial sequences were excluded from this study. The FASTA file of all the complete genome sequences was downloaded and included in the study. The sequences were imported into BioEdit 7.2 software to conduct multiple sequence alignment analyses (MSAA) [31]. Furthermore, the result from the MSAA was uploaded on MEGA 11 software, and a phylogenetic tree was constructed. The evolutionary history of each virus was calculated using maximum likelihood reconstruction by the general time reversible model with a gamma distribution and proportion of invariable sites (GTR + I + G) as shown in Fig. 2, the most appropriate model based on the lowest AIC score with 500 bootstrap replicates [32].

### The role of socioeconomic and ecological factors in the spatial temporal distribution of rabies globally

Understanding the intricate interplay between socioeconomic and ecological factors is paramount in comprehending the global dynamics of RABV transmission. The multifaceted nature of these influences underscores the need for a holistic approach to rabies prevention and control. By addressing both the socioeconomic barriers to healthcare access and the ecological determinants of rabies persistence, we can pave the way for more effective interventions and ultimately work towards a rabies-free world [33].

### Socioeconomic factors

The socioeconomic landscape exerts a profound influence on the transmission dynamics of rabies. It encompasses a range of elements, including access to healthcare resources, cultural practices, and economic disparities [34]. Lower socioeconomic status often translates to reduced access to healthcare facilities, creating a significant barrier in the timely treatment and prevention of rabies. This limitation stems from financial constraints, lack of awareness, and inadequate healthcare infrastructure. As a result, individuals in lower socioeconomic strata face heightened risks of rabies transmission [33].

Moreover, in rural areas, socio-cultural practices play a pivotal role in shaping the likelihood of exposure to rabies. Close interactions with domestic animals and wildlife, often integral to livelihoods and sustenance, heighten the risk of transmission in these communities. Traditional practices that involve handling of potentially rabid animals can inadvertently facilitate the spread of the virus [35]. Economic disparities on a global scale directly impact the distribution of resources for rabies prevention. This includes availability of vaccines, immunoglobulins, and healthcare infrastructure. Regions with limited economic resources often grapple with insufficient supplies of critical preventive measures, contributing to higher incidences of rabies cases [36].

### Ecological factors

The ecological context encompasses a diverse array of elements, each playing a crucial role in shaping the transmission patterns of rabies. Environmental determinants such as elevation temperature, and land cover type are pivotal environmental determinants that significantly influence the prevalence and spread of rabies. Regions with favorable climates which includes higher environmental temperatures and abundant wildlife habitats create optimal conditions for the persistence of rabies among animal populations which heightens the risk of spillover events, potentially leading to human infections [36, 37]*.* Furthermore, dog population dynamics (the prevalence of rabies in dog populations, particularly in less economically developed nations), contributes significantly to RABV persistence. Inadequate management and control of dog populations lead to sustained circulation of the virus which perpetuates the risk of transmission both within the canine population and to humans, amplifying the overall burden of the disease [36]. The environment, economic conditions, and human behaviors collectively exhibit spatial variations that exert a direct impact on the transmission dynamics of rabies. Through extensive investigations into spatial patterns, researchers have uncovered the diversity in geographic, climate, and environmental attributes that influence the movement of reservoir species, consequently dictating the potential for virus spillover to new and vulnerable areas [35].

### Climate influence

Climate factors, most notably average temperature, emerge as crucial covariates in the spatial distribution of human rabies [33]. Changes in temperature patterns can lead to shifts in the geographic range of rabies, potentially exposing previously unaffected populations to the virus (reference). These alterations in climate conditions can have profound implications for the overall prevalence and distribution of rabies cases [33].

### Reservoir species behavior

Among the most critical reservoir species for RABV is the common vampire bat. Its distribution and spread are intricately linked to a range of ecological factors, including altitude, climate fluctuations, humidity levels, and seasonal variations. Variations in these ecological determinants directly influence the behavior and distribution of vampire bat populations, ultimately shaping the potential for rabies transmission to both livestock and humans [38–41]

### Policy and resource allocation towards rabies elimination

The World Health Organization (WHO) estimates 35 000 human deaths from rabies annually worldwide between 2000–2012. A more recent estimate suggests an increase to 59 000 annual deaths, with 40% of these fatalities occurring in children under the age of 15 [42]. The estimated financial burden of rabies is approximately US$ 8.6 billion per year, of which 54% is for productivity losses due to premature deaths, 37% is for treatment of humans bitten by dogs suspected of being rabid and only 2% is spent on dog vaccination and population control [7]. One key strategy in the pursuit of rabies elimination is the vaccination of dogs, which are the primary carriers responsible for transmitting the disease to humans. By focusing on dog-mediated rabies and implementing widespread canine vaccination campaigns, we can significantly reduce the incidence of this fatal disease in the human population, ultimately bringing us closer to the 2030 target of rabies elimination. Substantial evidence from modelling studies and empirical data indicates that vaccination of 70% of dogs will be sufficient to eliminate canine rabies. [43, 44]

### Unified response for rabies elimination

The road to rabies elimination globally, particular in the context of Africa, has historically been fragmented, with actions taken independently and without coordination. However, the urgency of eliminating rabies by 2030, necessitates a united front. Recognizing this imperative, the One Health approach has emerged as the cornerstone of the strategy. A zoonotic disease such as rabies, affecting both animals and humans would require collaboration between the human health, animal health, and environmental sectors is essential. In 2015, prompted by an international initiative to establish the objective of eliminating human deaths from dog-mediated rabies worldwide by 2030, the World Health Organization (WHO), the World Organization for Animal Health (WOAH), the Food and Agriculture Organization of the United Nations (FAO), and the Global Alliance for Rabies Control (GARC) formed a collaborative structure known as the United Against Rabies collaboration (UAR) to pursue this goal [45]. The United Against Rabies collaboration leverages existing tools and expertise in a coordinated way to empower, engage and enable countries to save human lives from this preventable disease. The WHO’s global strategic plan puts countries at the center with renewed international support to act [45]. The WHO’s global strategic plan places countries at the forefront of efforts to combat rabies, backed by renewed international support. It adopts a practical approach with three main objectives:

### Objective 1: Rabies elimination through effective tools

The objective centers on eliminating rabies by employing vaccines, medicines, tools, and technologies effectively. This includes mass dog vaccination campaigns and timely treatment of exposed individuals. This has been made possible by the establishment of a rabies vaccine bank by WOAH in 2012, providing cost effective and timely access to high quality vaccines. Eradicating dog- mediated human rabies requires preventing a dog from getting infected and adequate tackling of the infected dogs. Moreover, effective health promotion and sensitization of people on how to avoid the bites of rabid dogs, to get treated upon exposure, and to immunize animals to halt the RABV transmission cycle. Recent efforts on immunization by WAOH have been commendable. In 2022 alone, about 800,000 rabies vaccine doses were delivered in Africa by the WOAH vaccine bank. The beneficiaries of these units include Benin, Botswana, Côte d’Ivoire, Chad, Mozambique, Namibia, Nigeria, Togo, and Zambia [45].

### Objective 2: Innovation and impact measurement

Objective 2 can be achieved through effective policies, guidance, and governance. Harmonizing international recommendations, frameworks, and methodologies by encouraging human capacity building in countries; and ensuring the bolstering of data collection and availability for sound decision-making is also very crucial. Also, by encouraging the use of technology use and advancement in health care delivery, strengthening surveillance capacity, and building an integrated reporting system. Harmonized international standards and guidance are crucial for enabling regional organizations and countries to effectively pursue rabies elimination. Since 2018, the United Against Rabies (UAR) initiative, led by WHO and WOAH, updated six global standards to align with best practices in rabies prevention and control, employing a One Health approach. This effort is essentially built upon established tripartite mechanisms (WHO, WOAH, FAO) which have solid programs and guidelines to strengthen human, animal, and environmental health systems and design missions or expert protocols for individual Member nations for curating robust national disease strategies and plans, especially on emerging and re-emerging zoonotic diseases like on rabies [45].

### Objective 3: Commitment and resource sustenance

Objective 3 centers on sustaining countries' dedication to rabies elimination and securing the essential resources through multi-stakeholder involvement. This involves showcasing the outcomes of initiatives carried out within the United Against Rabies collaboration in national, regional, and global rabies elimination efforts. By demonstrating the effectiveness of these activities, it instills confidence in the feasibility of global elimination. Additionally, Objective 3 aims to actively involve countries, stakeholders, and development partners in the collective endeavor to eradicate rabies. Objectives 1, 2 & 3 will help to ensure that dog vaccination programs are effective and comprehensive, that vaccines and information reach populations at high risk of rabies, and that dog bite prevention strategies are widely implemented. A secondary aim of these objectives is to make available trained professionals in human and animal health and education alongside accurate and comprehensive rabies surveillance and program monitoring [45]. Currently, there are approximately 100 countries with endemic dog-mediated rabies, to achieve the goal of zero rabies death by 2030 the UAR proposed a three phased approach to break down this ambitious global goal of "Zero by 30" into manageable steps.

**Phase 1 (Start-up**) of the rabies elimination strategy is dedicated to establishing a robust foundation for action. The focus is on creating normative tools and structures to initiate the process of rabies eradication. This plan began in 2018 targeting countries with the highest potential for success, such as those where rabies is a priority disease, those actively engaged in rabies control, those with ongoing pilot projects, and those from which we can draw valuable insights for future implementation. About 29 countries are currently in this phase with a total financial requirement of $16.5 million [13]. A key aspect of Phase 1 involves the formulation of national and regional rabies elimination plans. The integration of rabies control into national programs is essential for ensuring country ownership and implementing a sustainable approach tailored to local conditions. The role of UAR in this phase is to provide support and facilitate the development of these national plans, fostering coordination at the regional level. This collaborative approach is critical as efforts are currently geared toward the global goal of "Zero by 30" in the fight against rabies [13].

**Phase 2 (Scale-up)** represents a substantial expansion to 52 more countries particularly rabies endemic countries, encouraging them to commit to rabies elimination [13]. Building upon the strong foundation established in Phase 1 and informed by our collective learning and experience. During this phase, support for countries involved in Phase 1 remains resolute, ensuring the ongoing advancement of budgeted and sustainable national programs. Concurrently, efforts are extended to numerous new countries, initiating a parallel process with them. The celebration of successes assumes a pivotal role in sustaining momentum and motivating more countries to become part of the collective endeavor [13]. The focus becomes increasingly centered on regional elimination plans, fostering collaboration among countries to develop and execute regional strategies as they collectively advance toward the ambitious goal of achieving "Zero by 30" in the campaign against rabies. This phase is expected to extend from 2021 to 2025 with a budget estimation of $21.1 million from the UAR [13]

**Phase 3 (Mop-up**) marks the involvement of the remaining countries in the quest to eliminate rabies, with ongoing support for the efforts of communities, nations, and regions. Lessons gleaned from phases 1 and 2 will be invaluable in guaranteeing the success of this ultimate phase as the remaining 19 countries intensify their efforts towards rabies elimination. We must leverage all the accomplishments from the preceding phases to transform the vision of "Zero by 30" into a concrete reality. Phase 3 should span from 2026 till 2030 and has been allocated a budget of $12.1 million [13]. The partners in the United Against Rabies collaboration are already working in synergy, alongside countries, to facilitate and inspire global change. Investing in our global strategic plan will expedite the expansion and execution of our united approach, ultimately transforming rabies elimination from a vision into a concrete reality. Achieving zero rabies cases will undoubtedly have a profound impact on the lives and well-being of children and families worldwide [13]

### Utilizing the one health approach: targeting the critical control points in the epidemiological triads

**Fig. 3:**
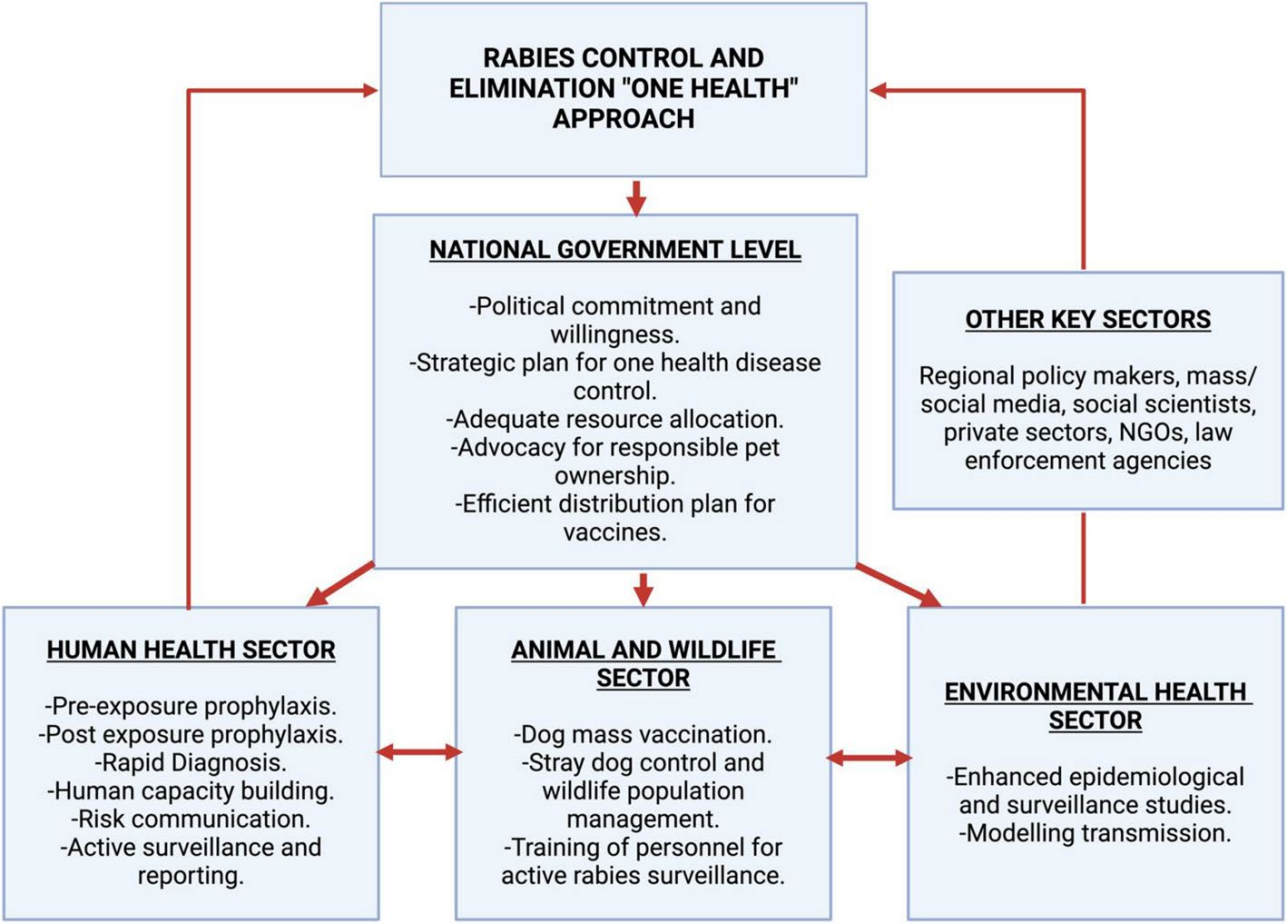
Conceptual framework of One Health approach at the national government level. A concerted effort between the animal and wildlife sector, human health sector, and environmental health sector, in collaboration with the national governments of various endemic countries in Africa is essential towards achieving effective rabies control and elimination in 2030. Adapted and modified from (Acharya et al., 2020). Created by Biorender.com

The One Health approach recognizes the interface and interdependence between the health of humans, animals, and their shared environment. The concept adopts the collaborative efforts across multiple disciplines and sectors at the local, national, regional, and global levels to heighten integrated disease surveillance, interventions, and sustainably balance and optimize health of people, animals, and ecosystems. Figure 3 describes a conceptual framework using One Health to control and achieve zero rabies agenda by 2030 [46]. Due to the multi-faceted complexity of rabies control and prevention, the one health concept is now being incorporated into the national one health strategic plans in various countries across the world to mitigate prioritized one health zoonoses such as rabies, and enhance swift outbreak management of the disease in both human and animals [46]. Furthermore, the adoption of the One Health approach is critical to the economies of low and middle income countries (LMICs) as this will reduce public health budgets associated with control of dog bites and PEP [47].

In 2015, the quadripartite of the World Health Organization (WHO), the World Organization for Animal Health (WOAH), the Food and Agriculture Organization of the United Nations (FAO), and the Global Alliance for Rabies Control (GARC) united to launch the zero dog-mediated human rabies deaths by 2030 [2] (WHO, 2022). These organizations have pledged alliance to support countries most affected by rabies to guide and speed up their actions towards the global strategic plan to end human rabies deaths mediated by dog bites by 2030 (Zero rabies by 2030). The key pillars of this planned actions include improved access to PEP, effective mass dog vaccination, upgraded capacities, tools or technologies for disease reporting and surveillance, community education and outreach, and to harness multistakeholder engagement to sustain commitment and resources [9]. Subsequently, in many African countries, these efforts have led to the prioritization of zoonoses and development of national One Health strategic plans, involving collaboration across sectors in medicine, veterinary medicine (both domestic and wildlife), and the environment, as well as other stakeholders in a view to allocate resources for the prevention and control of zoonotic diseases including rabies*.* Several developing countries such as Kenya, Vietnam, Ethiopia, India and Nigeria have identified and ranked rabies as top three One Health priority zoonotic diseases for targeted allocation of resources towards improving the disease surveillance, diagnostic capacity, data quality, risk communication, multisectoral partnerships, prevention and control [48–52].

For the first time, in 2017, the prioritization activities brought together over 61 experts across the human, animal, wildlife, and environmental spectrum in Nigeria. The experts prioritized 52 diseases using a modified semi-quantitative One Health Zoonotic Disease Prioritization (OHZDP) tool and ranked rabies as top zoonosis out of thirty-six zoonotic diseases based on the endemicity, epidemic potentials, severity, socio-economic impact, disease burden, and capabilities to prevent and control the disease in Nigeria [48]. The One Heath strategic approach has been implemented in several countries such as Bangladesh, the Philippines, Sri Lanka, Tanzania, Vietnam, and South Africa and have documented successes in the reduction of human rabies deaths [9]. For instance, Sri Lanka has been actively involved in implementing an integrated One Health approach towards rabies prevention, control and disease management. The country has achieved successful reduction of human rabies deaths through partnership with relevant stakeholders (such as the Public Veterinary Services, the Ministry of Health, animal health, wildlife and community based organizations, local governments, education and academia sectors, non-Governmental Organizations (NGOs), and law enforcement agencies), community engagement strategy to build awareness on rabies, sustained mass dog vaccinations, and accessibility to rabies PEP [2].

These efforts have contributed to the accomplishment story in this country by reducing human rabies deaths from 377 (2.9 per 100 000 population) in mid 1970s to 31 in 2021 [5], and a proof that rabies control by 2030 is achievable and sustainable in African countries if there is political will power, societal and financial commitments. Also, encouraging as many countries as possible in the African continent to develop and implement the national One Health strategic plans to control rabies will increasingly move us step closer towards the SDG 3 target of ending neglected tropical diseases, SDG 8 on achieving universal health coverage and saving an estimated 300 000 lives within 5 years and improving millions of lives across Africa [9].

Several key requirements and measures critical for successful and sustainable integrated control and elimination of dog mediated rabies may include establishing strong partnership between various national and international stakeholders from administrative units, private sector, NGOs and general communities [53]; improving capacity and enabling efficient policy decisions through the availability of integrated surveillance data or evidence supporting the design and implementation of the most cost-effective strategies [54]; integrating One Health surveillance systems that provides efficient and accurate data on dog and human population densities, rabies cases (suspected or confirmed and total infected populations), vaccinations of human and animals at a given space and time may be crucial in developing control frameworks and road maps for effective rabies control programmes [55]; well-coordinated surveillance, monitoring and data sharing systems among animal (including wildlife), human and environmental health sectors; increasing human accessibility to PEP and vaccination coverage in animals; improving community engagement, commitment, capacity building, and awareness towards rabies control especially in targeted vulnerable groups; strengthening capacities for prompt laboratory diagnosis; scaling up rabies research interventions and innovations; finally encouraging country government commitment and mobilization of sustainable investments and resources to eliminate rabies.

## Conclusion and recommendations

To mitigate the transmission of rabies on a global scale, and specifically in Africa, a multi-pronged approach is essential:

### Enhance access to healthcare resources

Implement targeted initiatives to improve healthcare infrastructure and access in underserved regions, ensuring timely treatment and prevention of rabies.

### Cultural sensitization and education

Conduct awareness campaigns to educate communities, particularly in rural areas, about safe practices when interacting with animals, reducing the risk of rabies transmission through cultural practices. Promote responsible dog population management: Implement comprehensive dog population control measures, including vaccination campaigns, sterilization, and responsible ownership practices to curb the spread of rabies.

### Monitor and address environmental changes

Implement robust environmental monitoring programs to track changes that may impact the distribution of reservoir species, and consequently, the transmission of rabies.

### Climate-resilient strategies

Develop strategies to adapt to climate change, considering its potential influence on the spatial distribution of rabies. By addressing these recommendations, we can significantly reduce the burden of rabies and move closer to achieving a world free from this devastating disease.

## Data Availability

Not applicable.

## Abbreviations

GARC: Global Alliance for Rabies Control
FAO: Food and Agriculture Organization of the United Nations
UAR: United Against Rabies collaboration
WAOH: World Organization for Animal Health
WHO: World Health Organization
